# On Deafness, Giddiness, and Noises in the Head

**Published:** 1897-09

**Authors:** 


**On Deafness, Giddiness, and Noises in the Head.** By Edward Woakes, M.D. Assisted by Claud Woakes. Fourth Edition. Pp. xvi., 224. London: H. K. Lewis. 1896.

This book, the title of which looks as if it had been designed to catch the eyes of patients rather than medical men, consists of a series of articles on some of the symptoms met with in ear diseases, arranged on no definite plan, but all harping on the one theme, that affections of the ear are caused by a peculiar disease of the nose, which the author is pleased to call "necrosing ethmoiditis." For the treatment of this affection we are referred to part 2 of this work, which is apparently not yet published. The author's pathology is as poor as his physiology, and we have little better opinion of his treatment so far as he here makes us acquainted with it.

